# Intussusception into the Colon

**Published:** 1849-02

**Authors:** 


### PATHOLOGY AND PRACTICE OF MEDICINE.

*Intussusception into the Colon.*—At the Pathological Society of London, Dr. Hare exhibited a specimen of this disease. A child, four years old, before going to bed, complained somewhat of soreness about the anus. On examining it, a few thread-worms were seen, as had frequently been the case before. On the following morning he got up and played with some other children in the house till between ten and eleven o'clock, when he suddenly complained of great pain in the lower part of the abdomen, to which he pressed his hands. He said he wanted to go to stool, and went two or three times in the ensuing quarter of an hour, but did not pass anything ; his eyes looked wild ; the pain continued severe, and about eleven o'clock he vomited all he had taken during the morning. Every half hour or less he continued to go to stool, but fruitlessly, except that about one o'clock, and passed a small lump or two of fœcal matter. The pain then for a while became apparently somewhat less than at first, and from about two till half past three p. m., he had some sleep.

At four p. m., he passed a clot of blood, and still continued to go to stool two or three times an hour, till a little after twelve on the Saturday. On many of those occasions he passed some pure dark-coloured blood, with a little slimy matter, the whole of which together, amounted, it was said, to eight or ten ounces. The pain in the abdomen remained intense ; he would only stay for a very short time in any one position, resting most frequently doubled up on his hands and knees, so as to relax as much as possible the abdominal muscles. He drank a great deal, and with avidity, but always vomited soon after. On the Saturday afternoon he went much less frequently to stool, and on the Sunday only twice, but the other symptoms, the pain and vomiting, remained much as before, up to the time when first seen by Dr. Hare (Monday afternoon.)

He obtained but little relief from the treatment adopted, and gradually sank, and died.

The *abdomen* presented externally the same aspect as during life.

On opening it, the two transverse prominences between the ends of the sternum and umbilicus were found to be caused, the upper one by the stomach, whose larger curvature was tilted unusually forwards and upwards ; the lower one by the *ascending* colon, which had become so displaced as to occupy nearly the position of the transverse colon, the caput coli being situated just above and but a very little to the right of the umbilicus. Both this portion of the colon and stomach were much distended with wind, as was also one coil of the small intestines. Scarcely any effusion into the peritoneum ; no lymph ; marked redness of most parts of the small intestines, especially where the different coils of them were in contact with each other. No redness of colon or of peritoneum covering the walls of abdomen. On examining the intestines it was discovered that there was a very considerable intussusception of ileum, and of the accompanying mesentery, through the ileo-cœcal valve into the colon, and that the appendix vermiformis was likewise carried through the valve ; two of the mesenteric glands close to the point of strangulation of ileum were enlarged. When the cœcum was opened, the ileum was found to have protruded into it to the depth of three and a half inches, so that at least seven inches of the small intestines must have been forced through the valve.—*Dub. Med. Press.*

